# Supplementary material for: Changes in Active Behaviours, Physical Activity, Sedentary Time, and Physical Fitness in Chilean Parents during the COVID-19 Pandemic: A Retrospective Study
Source: Int J Environ Res Public Health. 2022 Feb 6;19(3):1846. doi: 10.3390/ijerph19031846 (PMC8835301; doi:10.3390/ijerph19031846)
Supplement: Supplementary file 1 [file ijerph-19-01846-s001.zip › ijerph-1543495 -Supplementary.pdf]

Results

Table S1. Sociodemographic characteristics of participants.

	All (n = 201)	Fathers (n = 54)	Mothers (n = 147)	*p*-Value
Age (years)	41.39 ± 6.74	42.04 ± 6.67	41.15 ± 6.77	0.409
20–39 years	35.02 ± 3.79	36.14 ± 3.29	34.62 ± 3.90	0.109
40–59 years	45.86 ± 4.32	46.09 ± 5.21	45.78 ± 3.97	0.727
School type				
Public	121 (60.2%)	30 (55.6%)	91 (61.9%)	0.415
Private	80 (39.8%)	24 (44.4%)	56 (38.1)	
Number of children in scholar age				
One	90 (44.8%)	25 (46.3%)	65 (44.2%)	0.793
Two or more	111 (55.2%)	29 (53.7%)	82 (55.8%)	
Employment situation				
Normal, I moved to my job normally	31 (15.4%)	13 (24.1%)	18 (12.2%)	**0.042**
I have always worked in my home	8 (4.0%)	3 (5.6%)	5 (3.4%)	
I stayed in my home unable to work	16 (8.0%)	3 (5.6%)	13 (8.8%)	
I can work almost normally from my home	71 (35.3%)	21 (38.9%)	50 (34.0%)	
I can work for a few moments in my home	21 (10.4%)	4 (7.4%)	17 (11.6%)	
My job has been affected	21 (10.4%)	8 (14.8%)	13 (8.8%)	
Fired or my source income was suspended	8 (4.0%)	2 (3.7%)	6 (4.1%)	
My situation has not changed	24 (11.9%)	0 (0.0%)	24 (16.3%)	
I have been with prenatal/postnatal rest	1 (0.5%)	0 (0.0%)	1 (0.7%)	
MVPA (min/week)	575.53 ± 747.92	656.54 ± 732.38	545.78 ± 753.83	0.353
Physical fitness (score)	3.07 ± 0.93	3.43 ± 0.82	2.94 ± 0.93	**<0.001**
Sedentary time (min/day)	227.44 ± 186.42	245.83 ± 181.19	220.68 ± 188.47	0.398

MVPA: moderate-to-vigorous physical activity; Bold values indicate statistical significance.

Table S2. Baseline characteristics of active commuting, MVPA, and self-perception on the physical fitness by sex.

	**All (n = 201)**	**Fathers (n = 54)**	**Mothers (n = 147)**	***p*-Value**
Mode of commuting to work				0.111
Stay home	32 (16.7%)	4 (8.0%)	28 (19.7%)	
Active	19 (9.9%)	7 (14.0%)	12 (8.5%)	
Passive	141 (73.4%)	39 (78.0%)	102 (71.8%)	
Mode of commuting to home				0.560
Stay home	34 (17.7%)	6 (12.0%)	28 (19.7%)	
Active	19 (9.9%)	6 (12.0%)	13 (9.2%)	
Passive	138 (71.9%)	38 (76.0%)	100 (70.4%)	
MVPA (min/week)				
Public schools	619.49 ± 871.16	674.43 ± 890.24	601.37 ± 869.01	0.692
Private schools	509.05 ± 505.91	634.17 ± 485.59	455.43 ± 509.18	0.252
20–39 years	725.59 ± 979.18	854.77 ± 944.02	679.00 ± 995.04	0.474
40–59 years	575.53 ± 747.92	520.25 ± 516.01	451.28 ± 505.36	0.514
1 child	510.53 ± 655.41	763.72 ± 962.04	413.15 ± 464.34	**0.022**
≥ 2 children	628.23 ± 814.41	564.14 ± 450.43	650.90 ± 910.27	0.624
Physical fitness (score)				
Public schools	2.98 ± 0.88	3.38 ± 0.69	2.84 ± 0.90	**0.003**
Private schools	3.21 ± 0.98	3.48 ± 0.97	3.09 ± 0.97	0.096
20–39 years	3.18 ± 0.91	3.62 ± 0.76	3.02 ± 0.91	**0.007**
40–59 years	3.07 ± 0.93	3.29 ± 0.85	2.87 ± 0.95	**0.030**
1 child	3.08 ± 0.89	3.54 ± 0.57	2.91 ± 0.93	**0.002**
≥ 2 children	3.05 ± 0.96	3.32 ± 0.99	2.96 ± 0.94	0.077
Sedentary time (min/day)				
Public schools	214.38 ± 184.31	215.00 ± 170.27	214.18 ± 189.61	0.983
Private schools	247.19 ± 189.02	284.38 ± 190.53	231.25 ± 187.82	0.252
20–39 years	215.00 ± 166.18	182.05 ± 122.50	226.89 ± 178.73	0.281
40–59 years	227.44 ± 186.42	289.69 ± 202.70	216.28 ± 195.99	0.076
1 child	234.72 ± 188.73	238.80 ± 168.93	233.15 ± 197.03	0.900
≥ 2 children	221.53 ± 185.18	251.90 ± 193.90	210.79 ± 182.01	0.306

Mean and standard deviation or frequency and percentage; MVPA: moderate-to-vigorous physical activity; Bold values indicate statistical significance.

Table S3. Prevalence in active commuting "to work" and "to home," before and during pandemic according to mothers and fathers.

	Before	During	*p*-Value
	All		
Mode of commuting to work			**<0.001**
Stay home	32 (16.7%)	137 (72.9%)	
Active	19 (9.9%)	10 (5.3%)	
Passive	141 (73.4%)	41 (21.8%)	
Mode of commuting to home			**<0.001**
Stay home	34 (17.8%)	138 (72.6%)	
Active	19 (9.9%)	9 (4.7%)	
Passive	138 (72.3%)	43 (22.6%)	
	Fathers		
Mode of commuting to work			**<0.001**
Stay home	4 (8.0%)	29 (59.2%)	
Active	7 (14.0%)	2 (4.1%)	
Passive	39 (78.0%)	18 (36.7%)	
Mode of commuting to home			**<0.001**
Stay home	6 (12.0%)	30 (58.8%)	
Active	6 (12.0%)	3 (5.9%)	
Passive	38 (76.0%)	18 (35.3%)	
	Mothers		
Mode of commuting to work			**<0.001**
Stay home	28 (19.7%)	108 (77.7%)	
Active	12 (8.5%)	8 (5.8%)	
Passive	102 (71.8%)	23 (16.5%)	
Mode of commuting to home			**<0.001**
Stay home	28 (19.9%)	108 (77.7%)	
Active	13 (9.2%)	6 (4.3%)	
Passive	100 (70.9%)	25 (18.0%)	

Bold values indicate statistical significance.

Table S4. Influence of the COVID-19 pandemic in the variation of MVPA, physical fitness and sedentary time by sex of parents, school type, age group, and number of children.

	Sex of parents		***p*-Value**
	Fathers	Mothers	
Δ MVPA (min/week)	−257.46 ± 62.80 *	−239.94 ± 38.76 *	0.811
Δ Physical fitness (score)	−0.28 ± 0.08	−0.23 ±0.05 *	0.641
Δ Sedentary time (min/day)	98.22 ± 20.18 *	95.79 ±12.46 *	0.918
	School type		
	Public School	Private School	
Δ MVPA (min/week)	−286.81 ± 45.74 *	−210.59 ± 53.68 *	0.254
Δ Physical fitness (score)	−0.22 ± 0.06 *	−0.29 ± 0.06	0.428
Δ Sedentary time (min/day)	103.97 ± 14.70 *	90.05 ± 17.25 *	0.516
	Age group		
	20–39 years	40–59 years	
Δ MVPA (min/week)	−361.66 ± 54.31 *	−164.70 ± 45.69 *	**0.003**
Δ Physical fitness (score)	−0.36 ± 0.07 *	−0.18 ± 0.06 *	**0.025**
Δ Sedentary time (min/day)	87.95 ± 17.43 *	103.61 ± 14.66 *	0.462
	Number of children		
	1 child	≥2 children	
Δ MVPA (min/week)	−205.95 ± 53.10 *	−308.58 ± 47.94 *	0.128
Δ Physical fitness (score)	−0.28 ± 0.06 *	−0.26 ± 0.06 *	0.804
Δ Sedentary time (min/day)	124.35 ± 16.97 *	73.12 ± 15.32 *	**0.018**

Estimated marginal mean and error standard; MVPA: moderate-to-vigorous physical activity; ANCOVA models were adjusted by age, sex, and school type, excepting when variables were used to analyze stratified; MVPA: moderate-to-vigorous physical activity; Bold values indicate statistical significance; *Indicate a significance difference between own baseline value.

Table S5. Effect of the COVID-19 pandemic in the variation of MVPA, sedentary time, and physical fitness (score) by age group and number of schoolchildren category.

	G1	G2	G3	G4
Δ MVPA (min/week)	−250.09 ± 74.29 *	−472.62 ± 74.15 ø,§,*	−154.50 ± 69.45 ¥,*	−167.41 ± 57.83 ¥
Δ Physical fitness (score)	−0.35 ± 0.09	−0.37 ± 0.09	−0.21 ± 0.08	−0.16 ± 0.07
Δ Sedentary time (min/day)	85.12 ± 23.64 *	92.28 ± 23.60 *	160.02 ± 22.10 §,*	65.10 ± 18.41 ø

Estimated marginal mean and error standard; ANCOVA models were adjusted by sex of parents and school type; MVPA: moderate-to-vigorous physical activity; Δ Difference between before and during the pandemic; *Indicate a significance difference between own baseline value; Statistical difference with: ¥G2; øG3; §G4. G1: Group 20–39 years with 1 child; G2: Group 20–39 years with 2 or more children; G3: Group 40–59 years with 1 child; G4: Group 40–59 years with 2 or more children.
